# The foundations of the working alliance in assertive community treatment teams

**DOI:** 10.1186/s12888-021-03563-x

**Published:** 2021-11-10

**Authors:** M. van Haaren, S. de Jong, D. P. K. Roeg

**Affiliations:** 1 Department of Outpatient Psychiatry/Department of Forensic Psychiatry, GGzE Direct/De Woenselse Poort, GGzE, Eindhoven, the Netherlands; 2 Research Department, Lentis Groningen, the Netherlands; 3 Tilburg University, Tilburg, the Netherlands/Tranzo & Kwintes Supported Housing, PO Box 90153, 5000 LE Tilburg, Zeist, the Netherlands

**Keywords:** Working alliance, Assertive community treatment, Shared caseload, Multidisciplinary team, Assessment instrument

## Abstract

**Background:**

In this study, we aimed to identify and define the fundamental components of the working alliance in multidisciplinary (Flexible) Assertive Community Treatment teams with shared caseloads, in order to support their daily practice and further research.

**Methods:**

After reviewing the literature, concept mapping with professionals and clients was used to define the working alliance in (F) ACT teams. The resulting concept maps formed the basis for the working alliance assessment instrument, which was pilot tested with professionals and clients through cognitive interviews with a think-aloud procedure.

**Results:**

The study led to the development of a twenty five-item assessment instrument to evaluate working alliances in multidisciplinary teams (WAM) that was comprised of three subscales: bond, task/goal and team. Two different versions were developed for clients and professionals.

**Conclusions:**

The WAM instrument was developed to determine the quality of the working alliance in (F) ACT teams. Future research will focus on testing its psychometric properties and predictive value.

**Supplementary Information:**

The online version contains supplementary material available at 10.1186/s12888-021-03563-x.

## Introduction

In recent years, the care of clients with severe mental illness (SMI) has increasingly moved from institutional to community settings. Thus, several interventions have been developed to manage the care of clients with SMI in the community, including Assertive Community Treatment (ACT) and Flexible Assertive Community Treatment [1, 2] (FACT). Flexible Assertive Community Treatment is a variant of Assertive Community Treatment and is the dominant model used for community mental health care for clients with SMI in the Netherlands. Whereas ACT focusses on clients with SMI and acute needs through an intensive approach with caseload sharing, FACT integrates aspects of the ACT approach with individual case management and home visits for clients who are currently stable. Notably, FACT was developed because no effective treatment model existed for this group of clients with SMI. Whereas ACT teams serve clients with SMI who are in crisis or have the greatest care needs, FACT teams serve a more diverse population with variable care needs; therefore, they offer more continuity of care and ensure unique working alliances between caregivers and clients [3]. There are model fidelity scales available for both ACT and FACT teams [2]. Meta-analyses have shown that these types of community-based treatment programs are effective in facilitating symptomatic remission in homeless individuals with SMI and improve psychosocial functioning [4–8]. Despite decades of research into the working alliance, it is unknown how working in multidisciplinary teams with shared caseloads affects the working alliance and client outcomes.

### Working alliance research

It is well-established that the client-therapist relationship has implications for the effectiveness of treatment and interventions. Meta-analyses show a consistent relationship between the quality of the working alliance and the effect of psychotherapy on symptom severity [9–18]. There is no consensus on the definition of “working alliance”; however, most researchers adopt Bordin’s transdiagnostic definition of the alliance [19, 20]. Bordin formulated the working alliance as the degree to which the therapeutic relationship is engaged in collaborative work with purpose. Three domains were derived from his theory and can be measured using the Working Alliance Inventory [21]. These domains are the bond between therapist and client, the goals set for therapy and the tasks therapist and client agree upon completing to accomplish the goals.

For clients with SMI, a strong working alliance with their case manager or individual caregiver has been linked to positive outcomes, including greater treatment adherence, decrease in symptom severity, improved global functioning, greater quality of life and reduction in problem behaviours [22–26].

### Working alliance in the ACT/FACT paradigm

The primary function of community-based treatment programs such as ACT and FACT is that they offer outpatient care by a multidisciplinary team that shares the caseload. In multidisciplinary care, the working alliance becomes more complex and involves different types of relationships. The effects of these differences compared to the traditional therapist-client relationship were examined by several authors. First, according to clients, in (F) ACT the relationship between client and team may take the form of a more primary relationship and function as a hybrid of informal and formal roles [27]. Examples of informal roles include, but are not limited to, the role of confidant, acquaintance or social contact, examples of formal roles are those of psychiatrist, psychologist, social worker or nurse. Second, experience learns, community care may involve a wider array of more practical activities than non-community care [28]. Third, appliance of the model showed there can be more disagreement and conflict in the therapeutic relationship because the team serves both social control functions such as reaching out to non-compliant patients or patients who avoid care to ensure societal safety, as well as therapeutic and supportive functions [29]. Finally, professionals appreciate sharing the emotional and practical burden of treating clients with SMI with other caregivers [30]. In considering these multidisciplinary teams, several studies have indicated that the working alliance (measured with traditional assessment instruments) shows little to no effect on treatment outcome [31–36]. Furthermore, the few results that have been found have not been replicated and meta-analyses have not been conducted.

It is unclear why findings from studies involving clients and individual therapists do not appear to extend to clients and multidisciplinary care teams. Some authors hypothesize that this is a function of the instruments used for assessment of the working alliance. Based on this hypothesis, Klinkenberg et al. decided to supplement the way they evaluated the working alliance between clients with SMI and a case manager by adding items to the commonly used Working Alliance Inventory [35, 36], such as the number of case manager contacts and several client variables. However, they still did not find a relationship between the strength of the working alliance and treatment outcomes.

One challenge of measuring the influence of shared caseloads on the working alliance is the absence of an assessment instrument tailored specifically for use in multidisciplinary teams with shared caseloads. Therefore, in the current study we aimed to identify and define the key components of the working alliance in multidisciplinary teams with shared caseloads in order to develop an assessment instrument for use in daily practice and research.

## Methods

The development of the assessment instrument was divided into four stages: literature search, concept mapping, drafting of the assessment instrument, and pilot testing and instrument finalization. Ethical approval was granted by the Research Ethics Committee of GGzE (registration number IMBB/2017022). The concept map sessions and the pilot testing were conducted in Dutch. The concept maps and assessment instrument were translated to English by the authors for publication.

### Stage 1: literature search

The literature was comprehensively reviewed for working alliance assessment instruments and for studies regarding the relationship between working alliance and treatment outcomes for (F) ACT clients with SMI. The search terms were as follows: ‘working alliance’, ‘working relationship’, ‘therapeutic alliance’, ‘therapeutic relationship’, ‘helping alliance’, ‘alliance’ AND ‘instrument’, ‘questionnaire’ AND ‘case management’, ‘assertive case management’, ‘flexible assertive case management’, ‘community mental health care’, ‘severe mental illness’ AND ‘(treatment) outcome’, and ‘recovery’. Articles cited by the identified studies were also reviewed for inclusion in this study. The topic, target population, and method of analysis for each study were recorded to identify common themes. .

### Stage 2: concept mapping

Two concept mapping sessions were organised to identify the key components of the working alliance between clients and (F) ACT teams. Concept mapping is a mixed qualitative-quantitative participatory approach that results in a graphical representation of the concepts, the way these concepts are organised and their relative importance [37]. Concept mapping has previously been used in FACT research to measure quality indicators [38, 39]. Both sessions were led by the first author and the scenarios for the sessions were written by the first author with feedback from the third author.

The first concept mapping session was conducted with team members of (F) ACT teams from GGzE (a large institution for mental health care in Eindhoven and surrounding cities, a region in the south of the Netherlands) and national (Dutch) experts in the field. The second session was conducted with clients and former clients of (F) ACT teams and peer support workers who received care from one of the teams in the Eindhoven region. GGzE has eight FACT teams which are regularly audited by a certification board (CCAF) and one ACT team. Each FACT team has a caseload of approximately 200 clients with SMI. In total, approximately 12,000 clients are cared for by GGzE each year.

Flyers and information letters were made and distributed among the teams to recruit participants. The care professionals provided clients with information letters; interested clients were contacted by the researchers with further information. After a reflection period, clients could agree or decline to participate. Professionals were also informed of the study through oral presentations in team meetings by the first author. Experts in the field were contacted by email. Information about the study was also provided to a client coordination centre visited by clients, former clients and peer support workers. Finally, information was posted on a website that promotes research at GGzE (www.ggzei.nl). Two field experts and 13 GGzE mental health care professionals participated in the first concept mapping session. Five current clients, one client in training to become a peer support worker and one former client participated in the second concept mapping session. A purposive sampling procedure was used to generate maximum variation in responses. Both groups were diverse in terms of gender, age, level of education and years of experience with multidisciplinary outpatient teams with shared caseloads (Table 1). In the client and peer support worker concept mapping session we guarded against perceived coercion by emphasizing that the researchers were independent of the (F) ACT teams the participants receive(d) care from and that all answers would be treated confidentially.

**Table 1:** Characteristics of the concept map session participants

Professionals (*N* = 15)	
Gender	8 females, 7 males
Age, mean (range)	45.2 years (34–59 years)
Profession	Psychiatrist (1)
	Clinical psychologist/psychotherapist (2)
	Mental health care psychologist (2)
	Case manager (4)
	Social psychiatric nurse (3), in training (1)
	Director of FACT certification board in the Netherlands (1)
	Professor specialized in community care (1)
Mean number of years of experience working in (F) ACT teams (range)	8.4 years (1–26 years)
Clients and former clients (*N* = 7)	
Gender	4 males, 3 females
Age, mean (range)	41.4 years (26–56 years)
Level of education	3 secondary vocational education, 2 BSc, 1 MSc, 1 primary education
Living situation	7 independent living
Employment status	3 paid work, 3 no work or daily occupation services*, 1 daily occupation services
Mean number of years receiving care from (F) ACT teams (range)	3.2 years (1–8.5 years)

* Daily occupation services include volunteer work, spending time in an activity centre, and informal care

At the start of each session all participants completed informed consent forms and provided personal demographic information. The sessions then followed a written scenario based on literature regarding concept mapping [37, 40–42]. The goal of the study was explained, after which the generation of statements started. All participants were given a piece of paper with the agenda for the day on one side and the focus of the meeting and the definition of a working alliance on the other side. The focus of the meeting and the definition of a working alliance were also written down on a whiteboard in view of all participants. The definition of a working alliance was formulated as broadly as possible to minimize influence from the researchers. Specifically, we defined a working alliance as ‘the relationship between a professional/treatment team and the client who receives care from this professional/team’. The focus of the concept map session was formulated as follows: ‘the following topics are important in the working relationship between clients and a team that consists of multiple disciplines and caregivers who are all involved with the client and who also visit the client at home’. During the brainstorming, participants were asked to think outside of the box and consider all possible elements of care, including relationships and treatments, that could possibly influence the working alliance.

After one hour, the participants were asked to individually sort their statements regarding the working alliance into categories and to rate them based on their importance using a five-point Likert scale ranging from ‘not very important’ to ‘very important’. Ariadne was used to analyse the data [43]; first, a binary symmetric similarity matrix was computed for each participant. Subsequently, the software calculated the similarity between any two statements in the same pile to create an aggregated group matrix. A high value in the group matrix indicates that many participants grouped those statements together and implies that the statements are conceptually similar in some way. The aggregated similarity matrix was used as the input for a principal component analysis that translated the correlations between statements into coordinates in a multidimensional space. Subsequently, cluster analysis was used with the coordinates to further classify the statements and group statements that were similar into clusters [41, 42]. During the final phase of the concept mapping session, the participants were involved in interpreting the concept maps in a structured group discussion.

Each concept mapping session took approximately 4 h. Professionals were allowed to register the session as work time, and all travel expenses were covered for both groups. All participants received a box of chocolates after the session.

### Stage 3: development of the assessment instrument

The two concept maps formed the basis for the construction of the assessment instrument. The first step in designing the assessment instrument was the identification of larger domains common to both concept maps and integration of the concepts. The interpretation of the participants was used to guide this task and further built upon by the researchers.

The second step was searching for the specific statements within the identified domains that were considered the most important by both clients and professionals. To this end, an overview of the domains including the underlying concepts from each of the concept maps and the matching statements was compiled; the statements were then categorised by their priority score. When generating items for the assessment instrument, a differentiation was made between statements from the concept map sessions with importance scores from 3.5 to 4, 4 to 4.5 and 4.5 to 5. All statements that were scored as 4.5 or higher were included in the preliminary item list. The statements were rewritten into items for the assessment instrument.

The third step was the construction of the assessment instrument. Visual Analogue Scales (VAS) were chosen as the response scales, ranging from totally disagree to totally agree and from very unimportant to very important. VAS scales were used because of their relative ease of use and their sensitivity to small differences in scores [44–46]. Participants were asked to rate their degree of agreement with each item as well as their perception of the importance of each item.

All included items were formulated for a client version and a professional version of the assessment instrument. An introduction to the assessment instrument and an open-ended question that asked the participant if the assessment instrument lacked important details of the working alliance were also added. The items were randomised to prevent items from the same domain being scored in a row to avoid influencing scores because of priming or bias.

In the final step, the assessment instrument was reviewed by the second and third author and a registered nurse who was not involved in this study.

### Stage 4: pilot testing & cognitive interviews

The clients and professionals that participated in the concept mapping sessions and clients who volunteered for the concept mapping sessions but were unable to attend were contacted through email and invited to participate in testing the assessment instrument. Six professionals attended both the concept mapping session and the pilot testing. Five clients participated in the pilot testing, including two former clients who did not participate in the concept mapping sessions. Two gift certificates (€10) were raffled among the participants (one in each group). Participants differed with respect to gender, age, level of education and years of experience in/with (F) ACT teams (Table 2). Participants were interviewed by the first author following a think-aloud procedure in which they were asked to state all their thoughts while reading and responding to the instrument. Advantages of cognitive interviews with a think-aloud procedure over other forms of testing questions such as expert review and behaviour coding are its usefulness in identifying problems with questions and its ability to explicitly assess the participants ability to comprehend, recall, judge and respond to the questions. Also, this method is particularly helpful in assessing ambiguities that come up during the cognitive process of answering a question [47, 48]. Part of the instruction to participants was as follows: ‘I want to ask you to read this questionnaire and verbalize your thoughts out loud. I’m not necessarily interested in your answers, but I would like to understand how you get to your answer and which problems and inconsistencies you come across in your thought process. [ …] This is not a test of your skills, but of the questionnaire. You can be open in your critique of the questionnaire; I want to know if there is anything wrong with the questions and if so, what. There are no right or wrong answers’. Following the instructions, the interviews started with a small exercise in which participants were asked to visualize their house and recount what they see to the interviewer.

**Table 2:** Characteristics of the cognitive interview participants

Professionals (*N* = 6)	
Gender	4 females, 2 males
Age, mean (range)	42.5 years (38–49 years)
Profession	Clinical psychologist/psychotherapist (2)
	Mental health care psychologist (1)
	Casemanager (1)
	Social psychiatric nurse (2)
Mean number of years of experience working in (F) ACT teams (range)	6.8 years (3–10 years)
Clients and former clients (*N* = 5)	
Gender	3 males, 2 females
Age, mean (range)	43.4 years (34–56 years)
Level of education Living situation	4 secondary vocational education, 1 BSc 5 independent living
Employment status	2 daily occupation services*, 2 no work or daily occupation services, 1 paid work
Mean number of years receiving care from (F) ACT teams (range)	5.4 years (1.5–11 years)

* Daily occupation services include volunteer work, spending time in an activity centre, and informal care

Participants were asked about several topics related to the assessment instrument including the introduction, individual items, scoring of the items, and the use of the VAS rating scale. All interviews were recorded and additional notes were made by the first author. The recordings were transcribed and analysed by the first and third author to reveal general strategies for answering the questions and difficulties with particular questions. All feedback from the interviews was summarized for each item and items were subsequently adjusted in response to the feedback in consultation with the third author.

## Results

### Stage 1: literature search

The literature review revealed that most working alliance assessment instruments differentiate between the perspective of the client and that of the caregiver [49]. Given that the professional’s experience of the relationship can differ from that of the client, it is valuable to have two different versions of the assessment instrument-one for professionals and one for clients [26, 49, 50].

The hypothesis that arose from the literature search was that clients of outpatient, multidisciplinary teams with shared caseloads value certain aspects of the working alliance more than other aspects, and that this difference is both important and personal. The first draft of our assessment instrument therefore addressed both the degree to which professionals and clients agreed with the statements and the importance of these statements to the individual.

### Stage 2: concept mapping

**Fig. 1:**
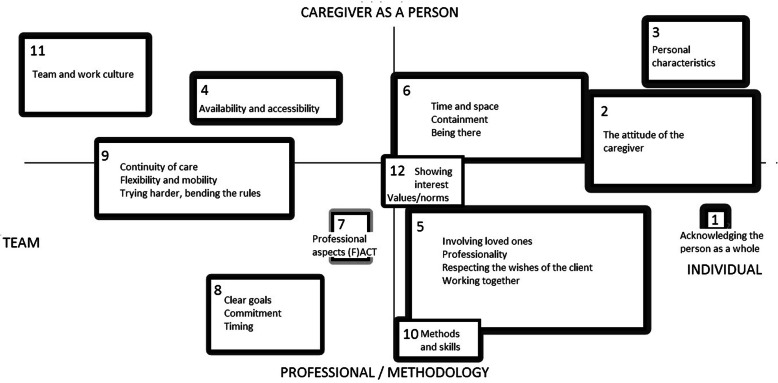
Concept map from the session with professionals and experts (*N* = 15)

**Fig. 2:**
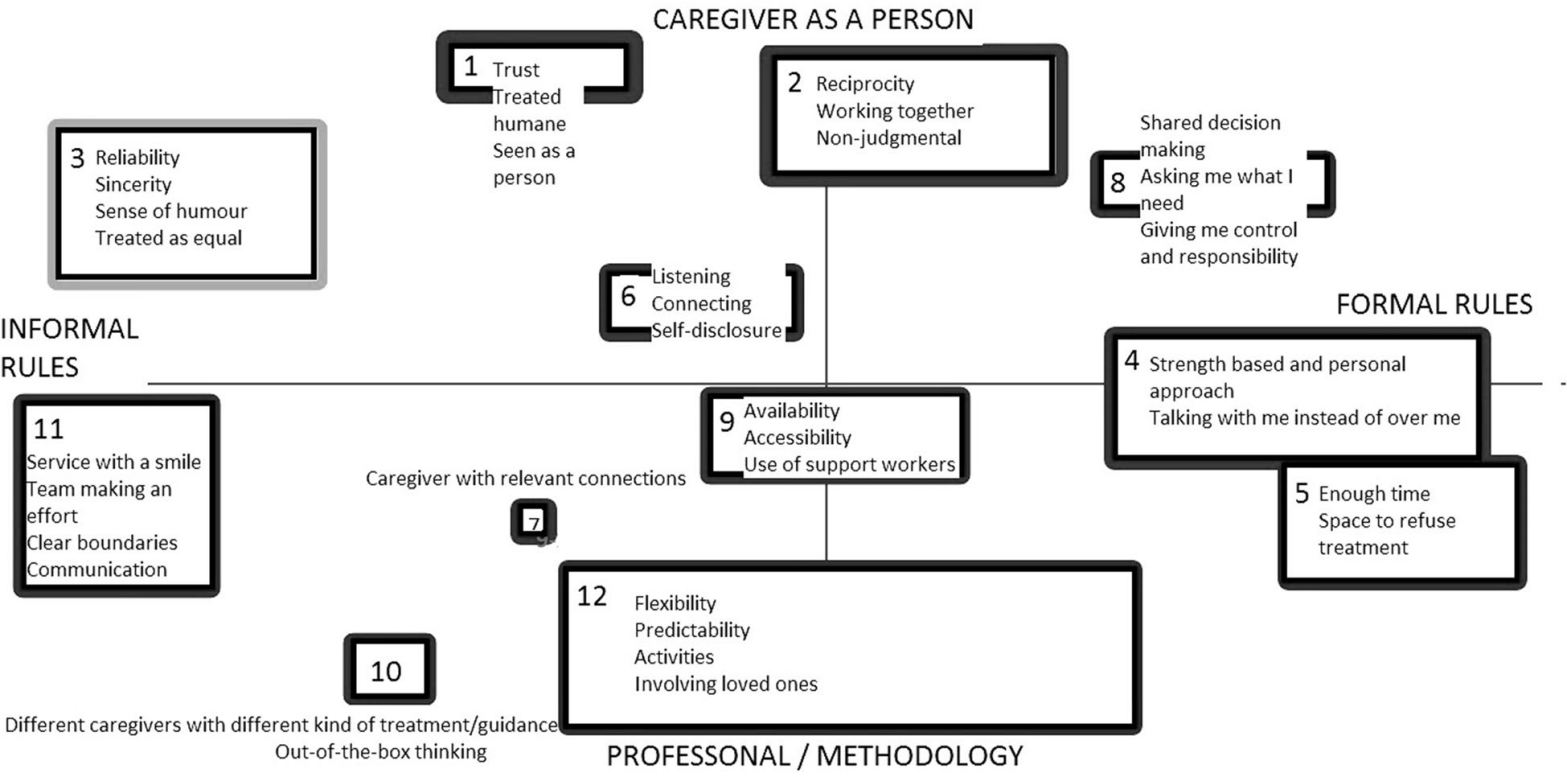
Concept map from the session with clients, former clients, and peer support workers (*N* = 7)

Figures 1 and 2 show the concept maps based on the 98 and 61 statements generated by professionals/experts and clients, respectively. The thickness of the lines surrounding the concepts indicate the mean importance score. The concepts were subsequently ranked in descending order of importance, with 1 indicating the most important concept.

In the concept map from the professionals’ session, ‘acknowledging the person as a whole’ was found to be the most important item. Its singularity shows that the participants did not link this item to other items; thus, it was recorded as a single concept.. The concept map from the clients’ session shows more diversity in the items underlying the concepts compared to the professionals’ concept map, which made it more difficult to capture the concepts in a single word or phrase (Fig. 2). Clients and professionals both found the statements regarding treating clients humanely to be very important.

In the concept map from the professionals’ session, the concepts move from team-based to more individual-based on the horizontal axis. The team-based concepts include aspects of team functioning such as team culture, flexibility and availability. Some of these concepts are tightly connected to the shared caseloads principle used in (F) ACT teams. The individual-based concepts include caregiver attributes such as attitude, skills, and professionalism. The vertical axis represents a continuum from the caregiver as a person to the caregiver as a professional in both concept maps. Thus, the top half of the concept map reflects personal characteristics, such as showing interest in the client, whereas the bottom half reflects professional aspects of the caregiver, such as professional skills.

Both clients and professionals made an explicit distinction between the professional and personal roles of the caregiver. Clients, however, did not make the distinction between caregiver teams and individual caregivers in the way that the professionals did; the horizontal axis instead shows a continuum ranging from more formal to more informal rules (Fig. 2). The formal rules section of the continuum includes elements such as shared decision making, involving the clients in treatment, and sharing responsibility. Conversely, the informal rules section of the continuum includes aspects of the caregiver-client relationship such as humour and sincerity.

### Stage 3: development of the assessment instrument

**Fig. 3:**
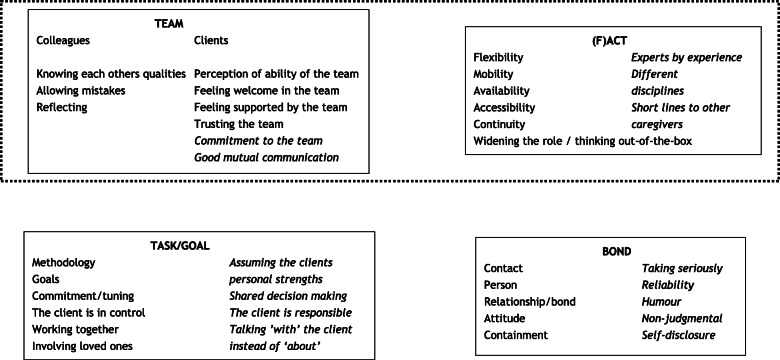
Concept model based on the concept maps. The black text represents concepts contributed by professionals, the italic text represents input by clients, former clients, and peer support workers

Combining the clusters of both concept maps led to the identification of several common concepts that were graphed in an overall concept model (Fig. 3). Although ‘team’ and ‘(F)ACT’ were two separate concepts in the concept maps, closer examination of the underlying data showed that both concepts were comprised of relatively few statements. The two separate concepts were therefore combined into one overarching domain (‘team/(F)ACT’). Three large common domains were identified: ‘bond’, ‘task/goal’ and ‘team/(F)ACT’. Since two of the three domains that remained conceptually overlapped with the WAI, they were labelled in accordance with the WAI subscale names of ‘bond’ and ‘task/goal’. The ‘bond’ domain concepts led to the formulation of nine assessment items, and the ‘task/goal’ and ‘team/(F)ACT’ domain concepts each resulted in eight assessment items.

Informal testing of the assessment instrument by the second and third author and an objective registered nurse led to small adjustments in the assessment items. These adjustments were mostly regarding complex words or sentences that seemed ambiguous or were unclear. Once adjusted, the first draft of the assessment instrument was comprised of 25 total assessment items.

### Stage 4: pilot testing & cognitive interviews

As a result of the pilot testing, several changes were made to the assessment instrument. First, the layout of the introduction was adjusted, font size was changed to enhance the visual appeal, and boldface type was employed to emphasize important elements. Second, feedback on the assessment items led to the removal of three items that evoked ambivalent reactions. The final open-ended question (i.e., if the participant felt important issues were missing from the instrument), led to the inclusion of two new items. The open-ended question was also rewritten for the final version. Minor corrections in language were made to several items and examples were added in some cases to improve comprehension of the item. The final assessment instrument contains 25 items, including the open-ended question regarding whether the questionnaire lacked assessment items for important aspects of the working alliance. The complete assessment instrument can be found in Additional File 1.

The importance rating was removed from the final version of the assessment instrument. Almost all participants showed a tendency to judge the items by their general importance rather than their personal importance. Instead a separate question was added to the instrument that asks participants to select the five items that are most important to their working alliance with a specific caregiver (client version) or client (professional version).

## Discussion

This study aimed to identify and define the fundamental aspects of the working alliance in (F) ACT teams. Concept map sessions were used to generate concepts for an assessment instrument. This led to the development and pilot testing of a 25-item instrument to assess working alliance in multidisciplinary teams (WAM).

The assessment instrument developed in this work differs from existing instruments in several ways. The gold standard in working alliance research is the ‘Working Alliance Inventory’, which differentiates between goal, task and bond dimensions and focuses on the working alliance between a client and an individual caregiver [21]. The assessment instrument developed here measures similar dimensions, but adds a new dimension specifically addressing aspects of the caregiving team and shared caseloads. Furthermore, while there is a the relatively new instrument designed for measuring the therapeutic relationship in community mental health care known as STAR, it does not take into account this team dimension [51]. Instead, instruments designed to measure working alliances with a team either measure working alliances with clinical or residential care providers [52] or measure a different concept, such as attachment [53]. Until now, there has been no instrument that measures the working alliance in multidisciplinary teams with shared caseloads [49].

The new assessment instrument proposed here integrates the differing perspectives of both clients and professionals. Although both groups agree on the importance of humane treatment of clients and differentiate between the professional and personal characteristics of the caregiver, professionals explicitly considered team-based factors as separate entity of the working alliance in (F) ACT teams. This makes sense since the added value of community-based treatment partly lies in the fact that these teams are able to offer flexible and continuous care. Professionals functioning in the team on a daily basis benefit from this arrangement continuously, whereas clients are less aware of the team’s organisational structure.

### Strengths

The assessment instrument developed in this work has several strengths. First, the instrument takes into account the perspectives of both the client and the professional. Several studies have shown there is only a low to moderate correlation between the working alliance scores of clients and those of professionals [12, 31, 32, 34, 51, 54–58]. Thus, the current study took both perspectives into account in the construction of the instrument and developed two versions of the instrument-one for the client and one for the professional.

A second strength of this work is that the assessment instrument is based on input from concept mapping sessions. The bottom-up development strategy was chosen to develop the instrument to ensure an innovative perspective on the working alliance, specifically for clients of (F) ACT teams. Almost all studies using existing instruments with small modifications found little to no significant relationship between the perceptions of the working alliance and the results of community care. Thus, a new approach to assessing the working alliance in this context that does not build on existing instruments is needed. The fact that several concepts that are widely used in working alliance research, specifically the bond, task and goals, emerged in the concept mapping sessions shows that there are some universal aspects of the working alliance; however, the assessment of these aspects must be complemented by the assessment of team-specific factors.

### Limitations

Despite these strengths, the limitations of this work also need to be considered. First, due to privacy legislation, participants had to be recruited through their caregivers, which may have contributed to selection bias. However, we did use purposive sampling strategies to ensure heterogeneity and recruited via the client coordination centre to maximize variety in responses. Given that the concept map sessions led to the identification of overarching concepts that can also be seen in existing working alliance assessment instruments, such as the Working Alliance Inventory [21], it is likely that some degree of generalizability can be presumed.

Second, as in most qualitative studies, the group of client participants was relatively small. It is possible this small group size affected the extent of the brainstorming and led to the identification of fewer and narrower concepts than a larger group would have. However, it was noticed during the concept mapping session with clients that some participants were much more vocal than others, which made it difficult for everyone to voice their opinions freely. Thus, a larger group may have led to greater challenges in participant concentration and participation.. Furthermore, the same general themes emerged repeatedly during the cognitive interviews, suggesting that conducting more interviews would not necessarily have led to different results. Future studies will test the psychometric properties of the instrument in a larger study population in order to strengthen the qualitative base of the assessment instrument. Finally, the cognitive interviews revealed a tendency to score items towards the ‘totally agree’ end of the VAS scale. Existing assessment instruments for working alliances tend to show a ceiling effect [36, 55, 57], which can be partially mitigated by reversing a number of the items. This reversal may counteract the ceiling effect; however, it also makes the assessment items more difficult to understand. Given the cognitive impairments that are often experienced by clients with SMI, using clear and direct language is preferable [59]. Additionally, VAS scales are better able to detect small differences in scores than Likert scales which may minimize the ceiling effect in this assessment instrument [44, 46, 60].

## Conclusions

This study aimed to develop a working alliance assessment instrument that measures specific characteristics of multidisciplinary outpatient teams with shared caseloads that care for clients with SMI. Concept mapping sessions were used to generate key concepts related to working alliances and the resulting instrument was pilot tested. This study led to the creation of a 25-item instrument to assess Working Alliances in Multidisciplinary teams (WAM). The WAM is the first instrument incorporating bond, task, goal and team aspects of the working alliance in this context and can be used in a wide variety of settings where client care is organised through multidisciplinary teams with shared caseloads.

In practice, caregivers can use the instrument to assess their working alliance with their clients and start a conversation with them about their experiences and values. This is important as caregivers may have different priorities than their clients, without either party realising this fact [60]. When clients and caregivers are more aware of their own perspective and that of the other party, possible pitfalls can be discussed and breakdowns in communication can be avoided. Although it is not always possible to influence the values that a client and/or caregiver consider the most important, discussing the limitations of care can strengthen the working alliance.

The WAM instrument can also be used in research settings to examine whether a qualitatively good working alliance leads to improved mental health care by (F) ACT teams and better patient outcomes. These outcomes may include features of both self-reported and clinical recovery, a higher perceived quality of life, lower care costs, or a better cost-benefit ratio for both clients and society. Finally, the WAM instrument may enable studies to focus on how to improve the quality of the working alliance through specific interventions, such as team supervision or training [20].

Future research will investigate the psychometric properties of the WAM instrument by assessing its reliability, investigating its factor structure, and establishing its predictive value and validity. Once validated, the WAM instrument will offer a new method to further improve care for clients with SMI.

## Supplementary Information

**Additional file 1.**

## Data Availability

Data and materials are available from the first author on reasonable request.

## Abbreviations

ACT: Assertive Community Treatment
FACT: Flexible Assertive Community Treatment
SMI: Severe Mental Illness
VAS: Visual Analogue Scale
WAM: Working Alliance in Multidisciplinary teams
