# Supplementary material for: The foundations of the working alliance in assertive community treatment teams
Source: BMC Psychiatry. 2021 Nov 10;21:559. doi: 10.1186/s12888-021-03563-x (PMC8582115; doi:10.1186/s12888-021-03563-x)
Supplement: Supplementary file 1 — Additional file 1. [file 12888_2021_3563_MOESM1_ESM.docx]

**WAM**

**Introduction of the client version:**

In this questionnaire we ask you about the relationship you have with the mental health care. ‘My caregiver’ is the caregiver you are in contact with the most. This will most likely be your case manager, but can also be a different team member, such as you psychologist or psychiatrist. ‘The team’ is the team your caregiver is part of, for example the FACT or ACT team.

On the next pages you see a number of statements. You can rate each of these statements on a scale. You do this by placing a X on the dotted line. It is about your estimate in the **past month**.

Examples:

If you kind of agree with the statement you place the X on the dotted line like this:

Totally disagree ………………………………………………………………X…………………….Totally agree

If you disagree with the statement you place the X on the dotted line like this:

Totally disagree ………X…………………………………………………………………………….Totally agree

You can place the X over the whole line, depending on the degree in which you agree with the statement.

On the last page we ask you which five statements you find most important in the working relationship you have with your caregiver and the team.

**Introduction of the professional version:**

In this questionnaire we ask you about the relationship you have with your client. ‘The team’ is the team you work in.

On the next pages you see a number of statements. You can rate each of these statements on a scale. You do this by placing a X on the dotted line. It is about your estimate in the **past month**.

Examples:

If you kind of agree with the statement you place the X on the dotted line like this:

Totally disagree ………………………………………………………………X…………………….Totally agree

If you disagree with the statement you place the X on the dotted line like this:

Totally disagree ………X…………………………………………………………………………….Totally agree

You can place the X over the whole line, depending on the degree in which you agree with the statement.

On the last page we ask you which five statements you find most important in the working relationship you have with this specific client.

| **Client version*** | **Professional version*** |
| --- | --- |
| **BOND** | |
| 1. My caregiver treats me equally. | I treat this client equally. |
| 1. My caregiver listens to me sincerely. | I listen to this client sincerely. |
| 1. I feel like my caregiver is involved with my care. | I feel involved with this client’s care. |
| 1. I experience my caregiver as open and honest. | I am open and honest to this client. |
| 1. My caregiver handles my emotions well. | I handle this clients emotions well. |
| 1. My caregiver treats me as a full human being. | I treat this client as a full human being. |
| 1. I trust my caregiver | This client trusts me. |
| 1. I gain hope from the contacts with my caregiver. | This client gains hope from his/her contacts with me. |
| 1. I feel safe with my caregiver and am not afraid to discuss difficult topics. | This client feels safe with me and is not afraid to discuss difficult topics. |
| **TASK/GOAL** | |
| 1. My caregiver asks me what I want regarding treatment and/or guidance and takes my wishes into account. | I ask this client what he/she wants regarding treatment and/or guidance and take his/her wishes into account. |
| 1. To the extent I agree to it, my caregiver works with my loved ones who matter to me (f.e. family members, partner, friends, acquaintances, other caregivers). | To the extent this clients wants me to, I work with the loved ones who matter to this client (f.e. family members, partner, friends, acquaintances, other caregivers). |
| 1. My caregiver focuses on what’s not going well, but also looks at what I can do well or what I am good at. | With this client I focus on what’s not going well, but I also look at what he/she does well of what he/she is good at. |
| 1. I co-decide about my treatment and/or guidance. | This client co-decides about his/her treatment and/or guidance. |
| 1. My caregiver takes into account the roles I fulfil or want to fulfil in society (f.e. parent, partner, child, friend, neighbour, employee, volunteer). | I take into account the roles this client fulfils or wants to fulfil in society (f.e. parent, partner, child, friend, neighbour, employee, volunteer). |
| 1. My caregiver tries his/her best to come to an agreement with me regarding the goals of treatment and/or guidance. | I try my best to come to an agreement with this client regarding the goals of treatment and/or guidance. |
| 1. My caregiver helps me with the things I want help with. | I help this client with the things he/she wants help with. |
| **TEAM and (F)ACT** | |
| 1. I feel welcome in the team. | This client feels welcome in the team |
| 1. The team offers me continuity in care: this means that there is continuity in appointments (f.e. with a different caregiver) and that there are no interruptions in care. | The team offers this client continuity in care: this means that there is continuity in appointments (f.e. with a different caregiver) and that there are no interruptions in care. |
| 1. I think the team likes working with me. | The team likes working with this client. |
| 1. The team puts in an effort for me. | The team puts in an effort for this client. |
| 1. The team visits me when I need them to. | The team visits this client when he/she needs it. |
| 1. The team is available for me when I need it. | The team is available for this client when he/she needs it. |
| 1. Within the team I can go to different caregivers for (different types of) treatment and/or guidance. | Within the team this client can go to different caregivers for (different types of) treatment and/or guidance. |

* Rate each item on the following scale:

Totally Totally

disagree agree

1. Do you feel like all the questions in this questionnaire together paint a good picture of the working alliance between you and your caregiver and the team? If your answer is no, which topics do you feel are lacking?
2. Which five statements do you find most important in the working alliance with your current caregiver and/or the team? It is not about which statements you find most important in general, but about the statements you found most important for yourself, **in the past month**. You can write down the numbers of these statements here.
